# Development and characterization of bladder acellular matrix cross-linked by dialdehyde carboxymethyl cellulose for bladder tissue engineering†

**DOI:** 10.1039/c9ra07965c

**Published:** 2019-12-18

**Authors:** Xu Peng, Pengfei Yue, Xiong Zhou, Li Li, Shuangshuang Li, Xixun Yu

**Affiliations:** College of Polymer Science and Engineering, Sichuan University Chengdu 610065 PR China yuxixun@163.com; Laboratory Animal Center, Sichuan University Chengdu 610065 PR China; Department of Biotherapy, Cancer Center, State Key Laboratory of Biotherapy, West China Hospital, Sichuan University Chengdu 610041 PR China; Department of Oncology, The 452 Hospital of Chinese PLA Chengdu Sichuan Province 610021 PR China

## Abstract

In order to address the disadvantage of rapid degradation and serious immune response of bladder acellular matrix (BAM) tissues in clinical application, in this study, oxidized carboxymethyl cellulose (DCMC) was developed to replace glutaraldehyde (GA), a most common synthetic crosslinking reagent in clinical practice, to fix BAM tissues for lower cytotoxicity. The aim of this work was to evaluate feasibility of DCMC as a crosslinking reagent for BAM fixation and developing DCMC fixed-BAM (D-BAM) tissues for tissue engineering. For the preparation of DCMC, the results showed that when DCMC was prepared using a specific concentration of sodium periodate solution (the mass ratio of NaIO_4_/CMC is 1 : 1) and a specific reaction time (4 hours), its cytotoxicity was the lowest and its fixation effect was better. The critical crosslinking characteristics and cytocompatibility of optimum D-BAM tissues were also investigated. The results demonstrated that DCMC-fixation (especially 30 mg ml^−1^ DCMC-fixation) not only formed stable cross-linking bonds but also preserved well the original ultrastructure of the BAM tissues, which simultaneously increased the mechanical strength and capacity of the enzymatic hydrolytic resistance. The DCMC-fixation could also reduce the expression of α-Gal in BAM tissues and preserve the useful growth factors such as GAGs, KGF and TGF-β in bladder tissues. In addition, 30 mg ml^−1^ D-BAM tissues had excellent cytocompatibility. Moreover, it could stimulate the secretion of PDGF and EGF from seeded bladder transitional epithelial cells (BTECs), which is a critical feature for further re-epithelialization. Its anti-calcification ability was also prominent, which is necessary in bladder repair. The present studies demonstrated that DCMC could be a potential biological crosslinking agent for BAM fixation due to its excellent crosslinking effects, and the D-BAM tissues were suitable to be used as a substitute for the bladder due to their resistance to enzymatic degradation, anticalcification and cytocompatibility.

## Abstract 2

To address the disadvantage of rapid degradation and serious immune response of bladder acellular matrix tissues in clinical application, oxidized carboxymethyl cellulose was developed to replace commonly used glutaraldehyde, to fix BAM tissues for lower cytotoxicity.

## Graphical abstract

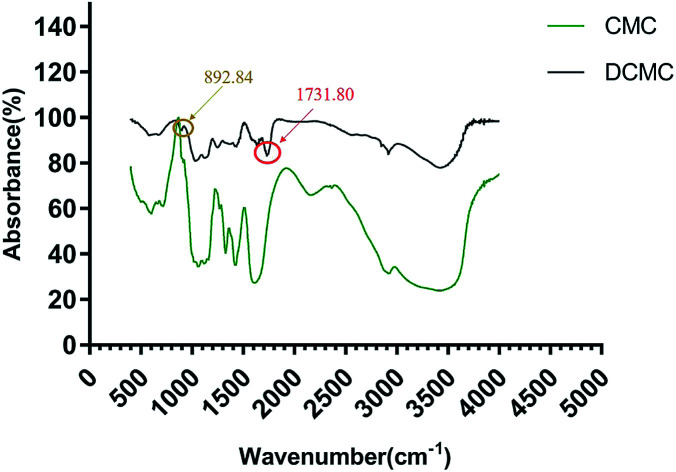

## 1. Introduction

A variety of congenital and acquired conditions result in bladder defects. Therefore, there is considerable demand for biomaterials that can be used as grafts for bladder reconstruction. There has been increasing interest in developing natural decellularised matrices from a variety of tissue derivations due to their unique advantage. Naturally derived, decellularized biomaterials usually contain structural molecules including collagen, elastin, fibronectin, growth factors, and others. All of these materials could orchestrate adherence, growth, migration, and differentiation of UCs, SMCs, endothelial cells, and nerve cells. Among these naturally derived biomaterials, BAM has been widely used to repair or replace bladder defects caused by illness or accident.^1^ BAM retains a suitable microscopic porous three-dimensional structure and contains a large number of extracellular collagens, fibronectin, and sulfated GAG. BAM also contains a large number of growth factors and cytokines, including platelet-derived growth factor BB (PDGF-BB), bFGF, keratinocyte growth factor (KGF), transforming growth factor-β (TGF-β), insulin-like growth factor (IGF), and VEGF,^2^ which can promote bladder cell migration, proliferation, and differentiation during the process of regeneration.

However, the natural bladder tissue is not stable. The instant degradation and the antigenicity presented in allogenic or xenogenic bladder tissues prevent these BAM from being preserved or being used directly in the clinic.^3,4^ Many techniques have been developed for preparing these decellularized biomaterials in order to overcome the disadvantages mentioned above, and provide suitable microscopic network structure for regeneration processes. Among them, the fixation of BAM by crosslinking agent can preserve its mechanical properties, reduce antigenicity, prevent enzymatic degradation, and maintain the original ultrastructure.^5,6^

Currently, some synthetic crosslinking reagents such as formaldehyde, glutaraldehyde, and polyepoxy compound have been widely applied in the fixation of decellularized biomaterials.^7,8^ However, the application of decellularized biomaterials fixed by synthetic crosslinking reagents in clinics is limited by side-effects like calcification, mismatched mechanical properties and high cytotoxicity.^9^ Carboxymethyl cellulose (CMC) is an important cellulose derivative. It is an anionic linear polymer cellulose ether widely used in food, textile, paper, paint, medicine, cosmetics, *etc.* Its excellent characteristics such as nontoxic, biodegradation and good biocompatibility suggest that CMC has potential suitable value in tissue engineering and medical applications.^10^ To address the side-effects of synthetic crosslinking reagents, we used sodium periodate to selectively oxidize the *o*-hydroxyl moiety on the C2 and C3 positions of the cellulose sugar ring to form dialdehyde carboxymethyl cellulose (DCMC) with a large number of multiple functional aldehyde groups which could be used as a crosslinking reagent. Our previous works have proved that DCMC was an effective agent in the fixation of porcine aortas.^11^ However, to the best of our knowledge, there is almost no literature report about investigation on the performance characteristics of BAM tissues crosslinked by DCMC.

In the present study, we attempt to assess feasibility of DCMC as a crosslinking reagent for BAM fixation and developing D-BAM for tissue engineering. The crosslinking characteristics, biomechanical properties, ultrastructure, enzymatic hydrolytic resistant ability and anti-calcification of D-BAM were investigated. The cytocompatibility of D-BAM and their bioactivity were also evaluated in cellular research.

## 2. Results and discussion

### 2.1 Assessment of DCMC

#### 2.1.1 FTIR analysis of DCMC

**Fig. 1:**
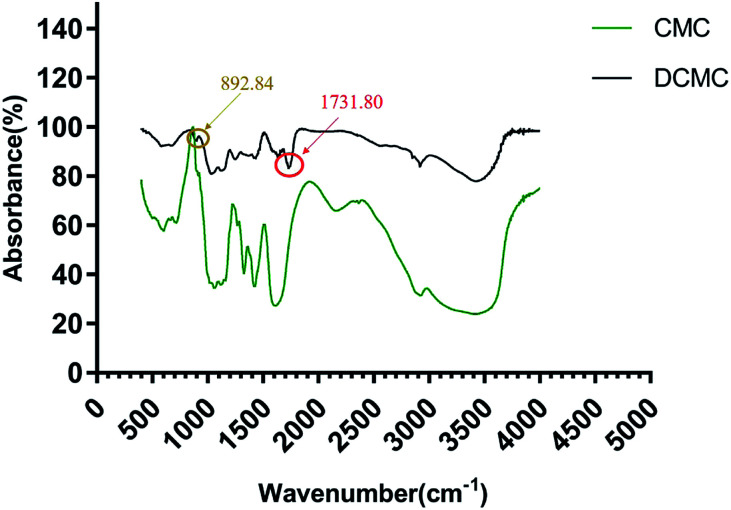
Infrared spectrum of CMC and DCMC.

The formation of dialdehyde groups of DCMC was verified by FTIR analysis. As shown in Fig. 1, the spectrum of DCMC showed a new characteristic peak at 1731.80 cm^−1^ that is the most characteristic band of C=O vibrations in aldehyde groups, which clearly indicated the formation of active aldehyde groups in DCMC. In the fingerprint region, a new infrared band at 892.84 cm^−1^ indicated the formation of hemiacetals between aldehyde groups and the hydroxyl groups of unoxidized residues.

#### 2.1.2 Cytotoxicity of DCMC

**Fig. 2:**
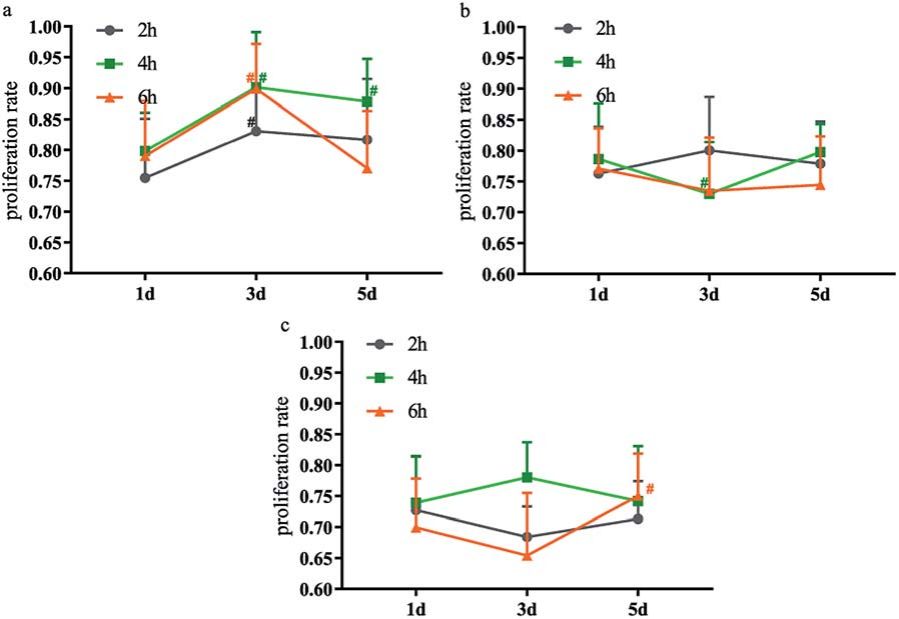
Effect of different NaIO_4_/CMC mass ratios and reaction time on cytotoxicity of DCMC ((a) NaIO_4_/CMC = 1 : 1; (b) NaIO_4_/CMC = 1 : 1.1; (c) NaIO_4_/CMC = 1 : 1.3), # means *p* < 0.05 compared with 1 d.

The cytotoxicity test results of DCMC with different OD generated by using different concentrations of sodium periodate solution and different reaction time suggested that RGR of L929 fibroblasts in various groups were between 75% and 99% (Fig. 2). Among them, the RGR of cells cultured in the medium drugged with optimum DCMC (o-DCMC) which was prepared using a specific concentration of sodium periodate solution (the mass ratio of NaIO_4_/CMC is 1 : 1) and a specific reaction time (4 hours) was the best. As we know, the shorter the reaction time for preparation of DCMC leading to incomplete reaction was, the bigger the molecular weight of DCMC was. On the contrary, the longer the reaction time or the higher the concentration of sodium periodates used in preparation of DCMC was, the smaller the molecular weight of DCMC was. DCMC with high molecular weight has low cytotoxicity and low crosslinking property, while DCMC with small molecular weight has high cytotoxicity and high crosslinking property. Therefore, DCMC used as crosslinking agent for fixation of BAM must have an appropriate molecular weight which could endow DCMC with low cytotoxicity and high crosslinking property. As mentioned above, the cytotoxicity of o-DCMC is the lowest in this study. Moreover, our researches also demonstrated that o-DCMC possessed good crosslinking property with molecular weight of 10 616 (ESI†). In view of this, we chose o-DCMC as the cross-linking agent for subsequent experiments.

The OD of synthesized DCMC was detected by hydroxylamine hydrochloride method which has been characterized to be reliable and accurate for the determination of aldehyde content. The measured OD of o-DCMC was 65.1%, which was also selected as the optimal oxidation degree for perfect crosslinking affect.

### 2.2 Characterization of BAM and D-BAM

#### 2.2.1 Residual DNA of BAM

**Fig. 3:**
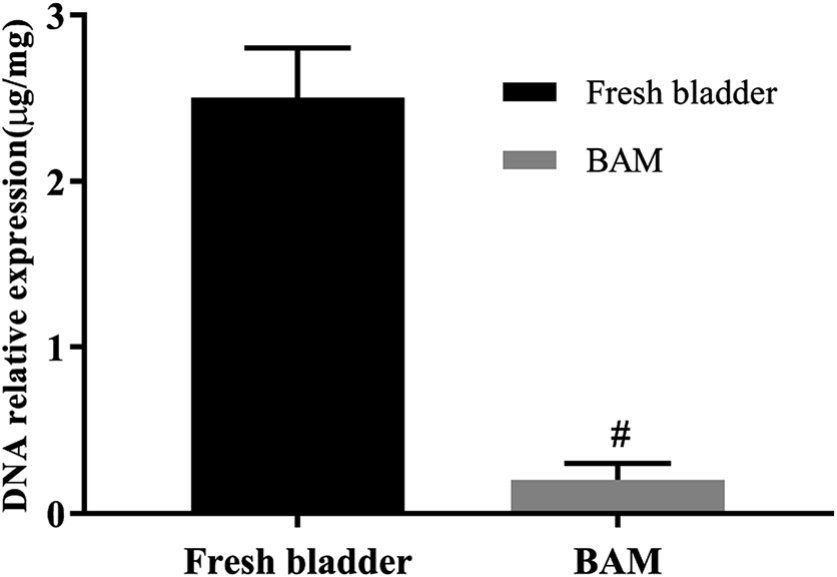
Residual DNA in porcine fresh bladder and BAM tissues. *n* = 3, means ± SD, # means *p* < 0.05 compared with fresh bladder.

Residual DNA is an important factor eliciting the potential adverse immune response for BAM tissues, and it is also considered as the cause of “inflammatory reactions” following the implantation of BAM scaffolds. Although the U.S. Federal Drug Administration does not regulate the limits for DNA in biological scaffold materials currently,^12^ we could expect that the lower the content of DNA in BAM tissues is, the better the BAM scaffold is. In this experiment, the DNA content of BAM tissues was significantly decreased by 92% (0.2 μg mg^−1^) after decellularization when compared to the fresh bladder tissues (2.5 μg mg^−1^) (Fig. 3). The DNA content was significantly reduced by decellularization and BAM tissues was suitable for follow-up study.

#### 2.2.2 Antigen and growth factors in BAM and D-BAM

**Fig. 4:**
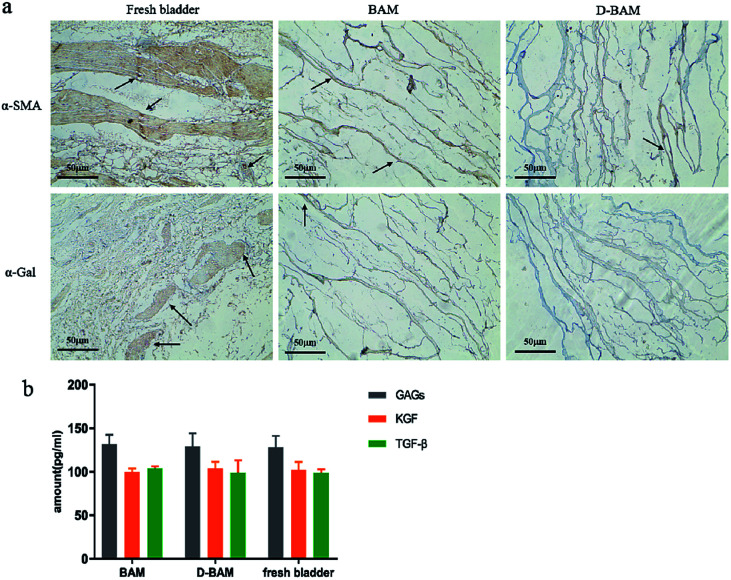
α-SMA, α-Gal expression (a); GAGs, KGF, TGF-β growth factor content (b) in fresh bladder, BAM and D-BAM (black arrows indicate the expression of α-SMA and α-Gal), bar = 50 μm.

In this chapter, besides the changes of antigen and growth factors in bladder tissues before and after decellularization, we also presented the effect of DCMC on the antigen and growth factors of BAM. α-SMA is specifically expressed in smooth muscle cells, and its expression level can be used as one of the criteria for determining the degree of clearance for smooth muscle cells.^13^ α-Gal antigen is widely distributed in various animal-derived tissues. α-Gal on heterologous biological tissues will bind to the naturally occurring anti-α-Gal antibody in the body, causing hyperacute immunity rejection reaction.^14^ The α-Gal-free feature can be also considered as a sign of efficient decellularization treatment (confirming the absence of cell membrane remnants). Therefore, reducing the potential immune risk of α-Gal, like the removal of cells, could also ensure the safety and effectiveness of the animal-derived biomaterial. α-SMA and α-Gal are strongly expressed in fresh bladder tissues, but their expression were very weak in BAM and D-BAM tissues (Fig. 4a). As to α-Gal, its expression in D-BAM tissues fixed by 30 mg ml^−1^ DCMC was more weak than that in BAM tissues. These results occurred due to the following aspects: the decellularization could remove the cells in fresh bladder tissues, and then greatly eliminated α-SMA and α-Gal. Moreover, the polypeptide, glycolipid and lipopolysaccharide in the BAM tissues are crosslinked to form insolubility macromolecule by DCMC. This could mask the α-Gal antigen determinant epitope of BAM tissues, and thus reduced the expression of α-Gal. These results suggested that the decellularization and DCMC fixation could greatly reduce the immunogenicity of fresh bladder tissues, and the prepared D-BAM tissues were suitable to be used as substitutes for the bladder.

GAGs play an important role in the cell matrix, not only to bind and protect a variety of endogenous biologically active factors, but to enhance water retention and serve as a substrate for cell growth.^15^ KGF can be used as a growth factor for the protection and repair of epithelial cells in various tissues, it plays a positive regulatory role after various forms of tissue damage, including bladder damage.^16,17^ TGF-β directly stimulates the expression of ECM proteins, including collagen, fibronectin and proteoglycans.^18–20^ Compared with fresh bladder tissues, the content of GAGs, KGF and TGF-β in BAM tissues and 30 mg ml^−1^ D-BAM tissues did not show obvious change (Fig. 4b). It indicated that the decellularization and DCMC fixation used in this paper did not damage the useful growth factor in bladder tissues. Wherefore, the advantage of D-BAM tissues for substitutes for the bladder was further confirmed.

#### 2.2.3 Ultrastructure of BAM and D-BAM

**Fig. 5:**
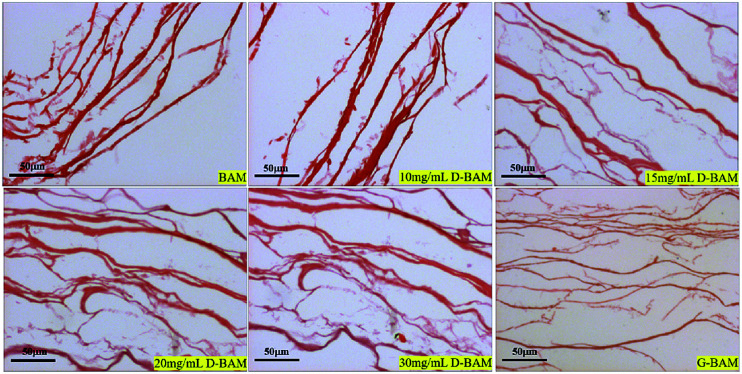
HE staining of BAM, D-BAM, G-BAM (bar = 50 μm).

**Fig. 6:**
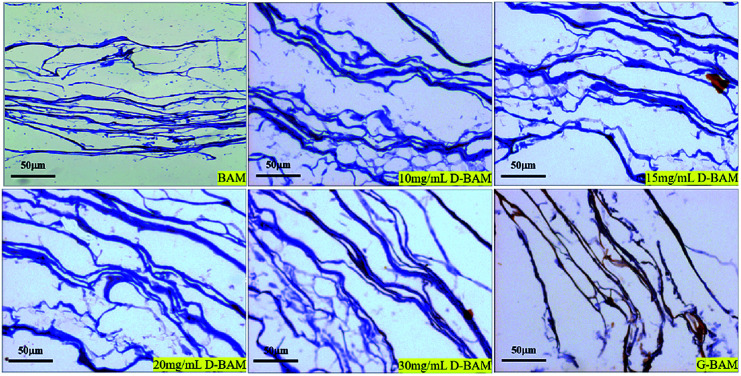
Masson staining of BAM, D-BAM, G-BAM (bar = 50 μm).

In order to observe the ultrastructure of the BAM and D-BAM, the bladder tissues were examined histologically by HE staining (Fig. 5) and Masson staining (Fig. 6). After decellularization, there were no residual cells in the fiber voids. The total framework of BAM tissues modified by various concentrations of DCMC all remained intact. Masson staining showed that the morphology of collagen fibers in the BAM tissues cross-linked with DCMC was well preserved, and the arrangements and orientations of them were as dense as the fresh sample. No fiber-entanglement or fiber-curling occurred. In the other hand, the collagen fibers in the BAM tissues fixed by GA became slightly stiff and broken. The main reason for the antigenicity of natural materials is the residual cellular components.^21^ The cellular components of BAM were completely removed by our decellularization method in this study, and then the antigenicity of BAM derived from cells was strongly reduced, the immune response elicited to BAM tissues was markedly diminished. Meanwhile, after fixation with DCMC, the structure of D-BAM tissues remained unchanged, and the good collagen fiber structure was largely preserved. This structure similar to that of fresh tissues, is suitable for the adhesion and proliferation of cells.^22,23^ Therefore, DCMC could be used as an effective cross-linking agent to cross-link bladder tissue.

#### 2.2.4 Fixation index

**Fig. 7:**
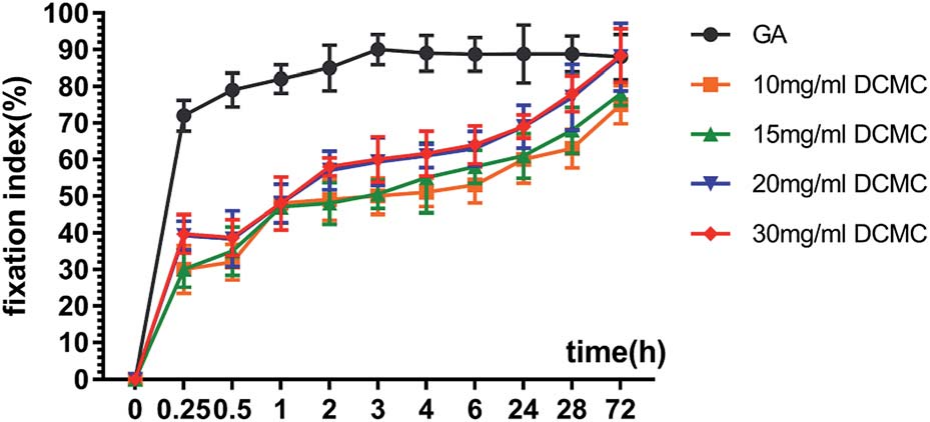
Fixation index of the BAM tissues fixed with GA and various concentration of DCMC.

The fixation index is used to evaluate the amount of free amino groups left in the biological tissues subsequent to the fixation and the crosslinking degree of the fixed biological tissues which is determined by ninhydrin (NHN) assay and reveals the percentage of amino groups reacting with aldehyde groups on the dialdehyde cellulose consumed to form stable cross-linking bridge structure.^24^ A higher fixation index often indicates a lower level of unreacted-free amino groups in the fixed biological tissues and a higher fixing degree in the fixed tissues.^25^ As shown in Fig. 7, for all tested samples, the fixation index increased with crosslinking-time elapsing, which indicated more stable cross-linking bridge formed within BAM tissues. However, the fixation index of the GA fixed BAM tissues increased more rapidly than DCMC-fixed ones, which demonstrated that the initial fixation rate of GA was faster than DCMC. After 3 h of fixation, the fixation index of GA-fixed BAM tissues nearly reached maximum (>90%), while DCMC-fixed BAM tissues' fixation index reached the comparable degree (89%) at the end of fixation process (72 h), which might due to scarce steric effect for small molecule-GA and it could react with free amino groups within BAM tissues more easily. Although a longer time was required for DCMC to reach the fixation index similar to GA, the ultimately crosslinking effect was comparable. As to the tested samples fixed by DCMC in different concentration, the fixation index of BAM tissues crosslinked with DCMC in concentration of 30 mg ml^−1^ were higher than other counterparts at any time points and finally reached 89.20% at the 3rd day. Therefore, 30 mg ml^−1^ initial concentration was selected as optimal concentration for DCMC fixation of BAM tissues.

#### 2.2.5 Biomechanical properties

**Fig. 8:**
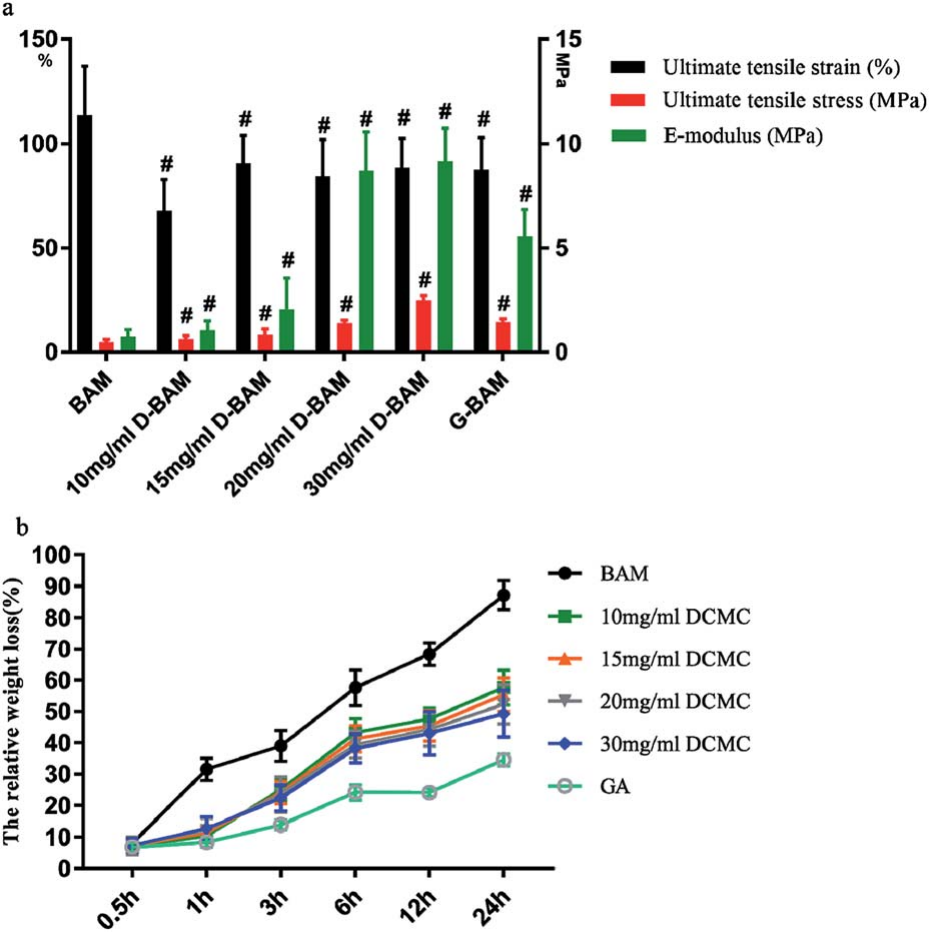
Mechanical property (a), the relative weight loss of BAM, GA fixed and various DCMC fixed BAM tissues during enzymatic degration *in vitro* at different time points (b). *n* = 3, means ± SD, # means *p* < 0.05 compared with BAM.

As a substitute with function during implantation for the bladder, suitable mechanical strength is required for crosslinked BAM tissues.^26^ In addition, the mechanical properties of the material is also a common indicator to distinguish between effective cross-linking of BAM tissues and the masking of the free amino group in the fixed BAM tissues.^27^ As shown in Fig. 8a, the mechanical strength of BAM tissues was significantly increased by DCMC-fixation. The values of “ultimate tensile stress” and “E-modulus” for G-BAM and D-BAM tissues were significantly higher than that of the unfixed BAM tissues, while the values of “ultimate tensile strain” decreased, which suggested unfixed BAM tissues a better elasticity. Therefore, we can obviously conclude the mechanical strength of BAM tissues increased when treated with crosslinking reagents. The ultimate tensile stress of D-BAM tissues increased with DCMC concentration increase and achieved maximum of 2.50 ± 0.21 MPa at 30 mg ml^−1^ DCMC, which was exactly in accordance with the result of fixation index. The E-modulus of D-BAM tissues was also concentration-dependent increasing in response to the increasing DCMC concentration and the highest level was achieved at 30 mg ml^−1^ DCMC. The increase of tensile stress of D-BAM also confirmed that effective crosslinking formed from reaction between the functional dialdehyde groups in DCMC and the amino groups in BAM tissues. This result also indicated that the BAM tissues were crosslinked effectively by DCMC and the masking of the free amino group in BAM tissues did not appear in this fixation processing. Although the unfixed BAM tissues presented a better elasticity, the D-BAM was more suitable to be used as a substitute with good mechanical strength for the bladder according to the mechanical behavior of bladder wall (repeatedly withstanding the force exerted by expansion and contraction of bladder).

#### 2.2.6 *In vitro* enzymatic degradation

In order to verify whether the fixed BAM tissues gained the performance of avoiding immediate degradation and extending storage time, evaluating its resistance ability against enzymatic degradation was absolutely necessary. Fig. 8b presents the relative weight loss of non-, GA- and DCMC-crosslinked BAM tissues during enzymatic degradation process *in vitro* at different time points. Under identical degradation situations in collagenase I buffers, the relative weight loss of all crosslinked BAM tissues was significantly lower than that of unfixed ones at any time points. With the prolongation of enzymatic proteolysis time, the relative weight loss of tested BAM tissues all obviously increased. However, the unfixed-BAM tissues were hydrolyzed more rapidly and extensively than fixed ones. After digesting for 30 min, the fresh BAM tissues were proteolysed by 8.35%, which was similar to the fixed BAM tissues. But the fresh BAM tissues were proteolysed by 80.52% after 24 h of proteolysis, while G-BAM tissues or 10 mg ml^−1^, 15 mg ml^−1^, 20 mg ml^−1^ and 30 mg ml^−1^ D-BAM tissues were proteolysed by only 34.53%, 56.38%, 54.45%, 52.69% and 50.83%, respectively. Meanwhile, the digestion rate was concentration-dependant decreasing in response to the increasing DCMC concentration and reached maximum at 30 mg ml^−1^, which was selected as the optimal concentration for fixation. The resistance against proteolysis of D-BAM, lies between fresh and G-BAM, were good in the early stage while later degraded properly with the prolongation of enzymatic proteolysis time to form some cavities that was beneficial to ingrowth of new tissues indicating the D-BAM tissues suitable for tissue engineering scaffolds. The remarkable resistance to proteolysis shown in the G-BAM and D-BAM tissues probably lied in being hidden of the collagen-cleavage sites by the reaction of crosslinking between the free amino groups in BAM tissues and aldehyde groups on GA or DCMC.^28^ Moreover, a dense layered network structure in fixed BAM tissues was formed, which was an obstacle for enzymatic penetration into fixed-BAM tissues.^29^

#### 2.2.7 *In vitro* anti-calcification

**Fig. 9:**
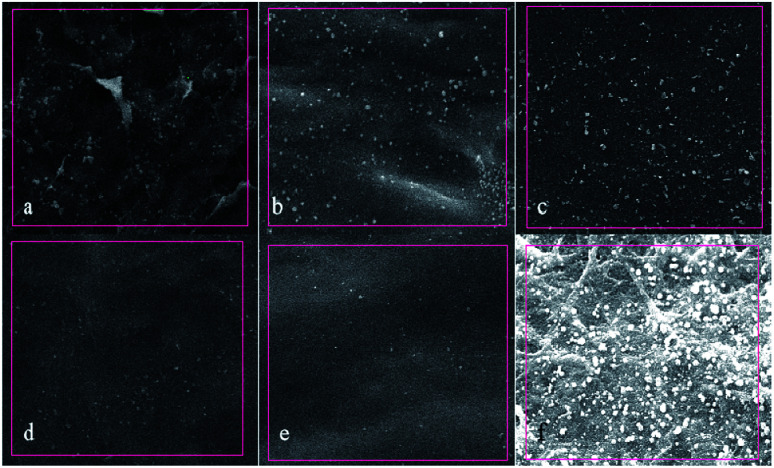
Surface calcification diagram of samples in each group under SEM (×1000). (a) BAM; (b) 10 mg ml^−1^ D-BAM; (c) 15 mg ml^−1^ D-BAM; (d) 20 mg ml^−1^ D-BAM; (e) 30 mg ml^−1^ D-BAM; (f) G-BAM.

Calcification can result in the stiffness and progressive deterioration of decellularized biomaterials, and then may cause the failure of transplantation of these materials.^29^ The calcification process has two main stages: nucleation and mineral propagation. Nucleation is the key to calcification of biological tissues.^30,31^ Previous studies on biomimetic mineralization *in vitro* have shown that anionic groups such as COO–, –COOH and –OH on the surface of the material attract calcium ions by electrostatic action, which may form hydroxyapatite minerals.^32^ Free aldehyde groups in the biological tissues is also a key factor leading to calcification. As shown in the EDS spectrum results (Fig. 9, Table 1) in this paper, a lot of white crystal particles resulted from the calcification process existed on the surface of GA-fixed BAM tissues and the accurate calcium weight percentage in GA-fixed BAM tissues was recorded as 0.55%; this suggested that GA fixation could notably accelerate the calcification process. The white crystal particles distributed on the surfaces of DCMC fixed BAM tissues were fewer and smaller, and the accurate calcium weight percentages in 10 mg ml^−1^ D-BAM, 15 mg ml^−1^ D-BAM, 20 mg ml^−1^ D-BAM and 30 mg ml^−1^ D-BAM tissues were respectively recorded as 0.43%, 0.36%, 0.25% and 0.1%; it indicated that DCMC, especially 30 mg ml^−1^ DCMC, had an inhibitory effect on calcification formation, which further prevented the progressive deterioration of BAM tissues and repair failure. The reasons for this may be attributed to the following: the large amount of aldehyde groups residual in G-BAM tissues could induce calcium crystal nucleus formation, which accelerated their calcification process. By contrast, the amount of aldehyde groups residual in D-BAM tissues was reduced due to the stabilization of cross-linking bonds between aldehyde groups in DCMC and free amino groups in BAM tissues by a reduction reaction with sodium borohydride following DCMC fixation, thus inhibiting their calcification process. Meanwhile, DCMC's anti-calcification result could be also caused by remaining the total framework of BAM tissues intact during DCMC crosslinking. Lastly, the residual aldehyde groups in DCMC could react with adjacent hydroxyl groups to form hemiacetals, which is also unbeneficial to calcification process.

**Table 1:** Determination of calcium content of samples in each group

Element	Weight percentage (%)					
	BAM	10 mg ml^−1^	15 mg ml^−1^	20 mg ml^−1^	30 mg ml^−1^	GA
Ca	0.48	0.43	0.36	0.25	0.10	0.55

### 2.3 Cytocompatibility of D-BAM

The effect of DCMC-fixed tissues on BTECs proliferation was examined *in vitro* by both the direct contact assay and indirect extraction assay. Extraction liquid was obtained after the sterilized samples being immersed and incubated in saline at 37 °C for 24 h in 5% CO_2_. Both tests were carried out using MTT assay. Generally, the optical density (OD) value from the MTT assay reveals the number of living cells in the cytocompatibility tests of samples; the value higher than the blank control indicates a higher cell viability or no cytotoxicity.

**Fig. 10:**
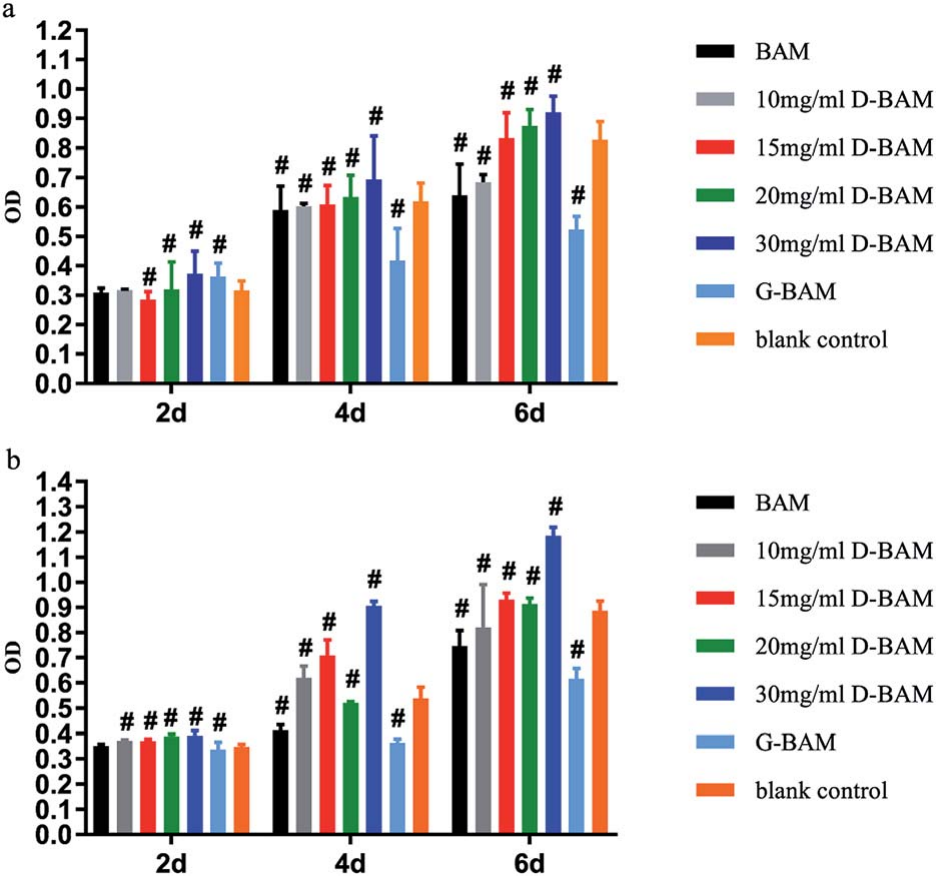
Cell viability of BTECs cultured in the extraction liquid of BAM, GA- and various concentration DCMC-fixed BAM tissues (a) and cell viability of BTECs cultured on these samples (b). *n* = 3, means ± SD, # means *p* < 0.05 compared with blank control.

As shown in Fig. 10, the results of both tests demonstrated that G-BAM tissues exhibited an obvious inhibition effect on BTECs proliferation while D-BAM tissues showed no cytotoxicity or even the promotion of BTECs growth and proliferation at 30 mg ml^−1^ of DCMC concentration. It indicated that DCMC is a novel BAM crosslinker with low cytotoxicity. The aldehyde groups in GA and DCMC all can react with the free amino groups of lysine, hydroxylysine or arginine residues within BAM tissues to form imino structure units. The high cytotoxicity of G-BAM tissues is mainly attributed to continuous leaching-out of the unreacted GA. Moreover, GA can easily diffuse through BAM tissue interstices and into BTECs cultured with them due to its low molecular weight. Therefore, GA is easier to react with proteins or polysaccharides on and inside the BTECs, and thus results to cell death. In contrast with GA, even though fixing BAM tissues through same mechanism, DCMC has lower cytotoxicity than GA because DCMC's high molecular weight hinders itself diffusing into BTECs or reacting with cellular component. Moreover, DCMC is derived from naturally occurring polysaccharide, which presents low cytotoxicity. The residual aldehyde groups in DCMC could react with adjacent hydroxyl groups in BAM tissues to form hemiacetals, which also significantly reduce the cytotoxicity of DCMC. In addition, the cytotoxicity of D-BAM was further eliminated by NaBH_4_ reducing major aldehyde groups and unstable Schiff base.^33^

**Fig. 11:**
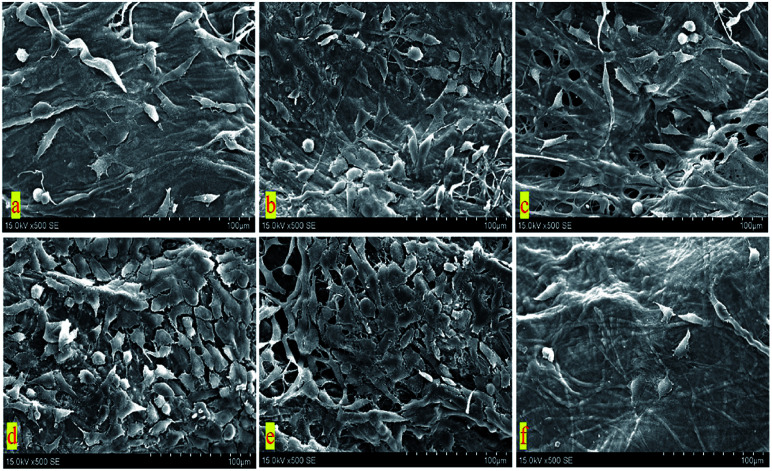
SEM of BTECs cultured on the surface of cross-linked BAM tissues (×500). (a) BAM; (b) 10 mg ml^−1^ D-BAM; (c) 15 mg ml^−1^ D-BAM; (d) 20 mg ml^−1^ D-BAM; (e) 30 mg ml^−1^ D-BAM; (f) G-BAM.

In order to further research the growth of BTECs on the surface of various D-BAM tissues, SEM was utilized to observe the morphology and spreading of BTECs on various tested specimens. As exhibited in Fig. 11, during the culture period of 4 days, a lot of spreading-BTECs were observed on the surface of the BAM tissues fixing by DCMC, especially BTECs on the surface of BAM tissues fixing by 30 mg ml^−1^ DCMC appeared to grow better and nearly reached the continuous cell layer. On the contrary, only very few rounded cells were observed on the surface of G-BAM tissue. These transmutative or sparse shaped cells suggested low viability or dead cells. It indicated that DCMC could provide a suitable microenvironment for BTECs attachment and growth. The result of SEM examination is in accordance with the consequence of MTT test.

### 2.4 Epidermal growth factor (EGF) and platelet derived growth factor (PDGF) protein secretion

As is well known, the epithelial regeneration are tightly regulated by some peptide molecules. Among them, EGF and PDGF are two attractive growth factors with possible ability to promote BTECs proliferation and attachment. Some studies have shown that EGF and PDGF could accelerate the growth of BTECs and promote the repair of bladder injury. Antoniades found that epithelial cells could express PDGF and PDGF receptors during regeneration.^34,35^ PDGF could stimulate epithelial cell proliferation through binding to PDGF receptors on the surfaces of cells. The other studies demonstrated that EGF could stimulate the uptake of tritiated thymidine in rat urothelial cells, and thus enhance the proliferation and migration of mouse urothelial cells.^36,37^ In addition, this growth factors not only regulate epithelial regeneration through paracrine matrix–epithelial interaction, but regulate epithelial regeneration through direct interaction of growth factors with cognate receptors in the epidermis.^38^

**Fig. 12:**
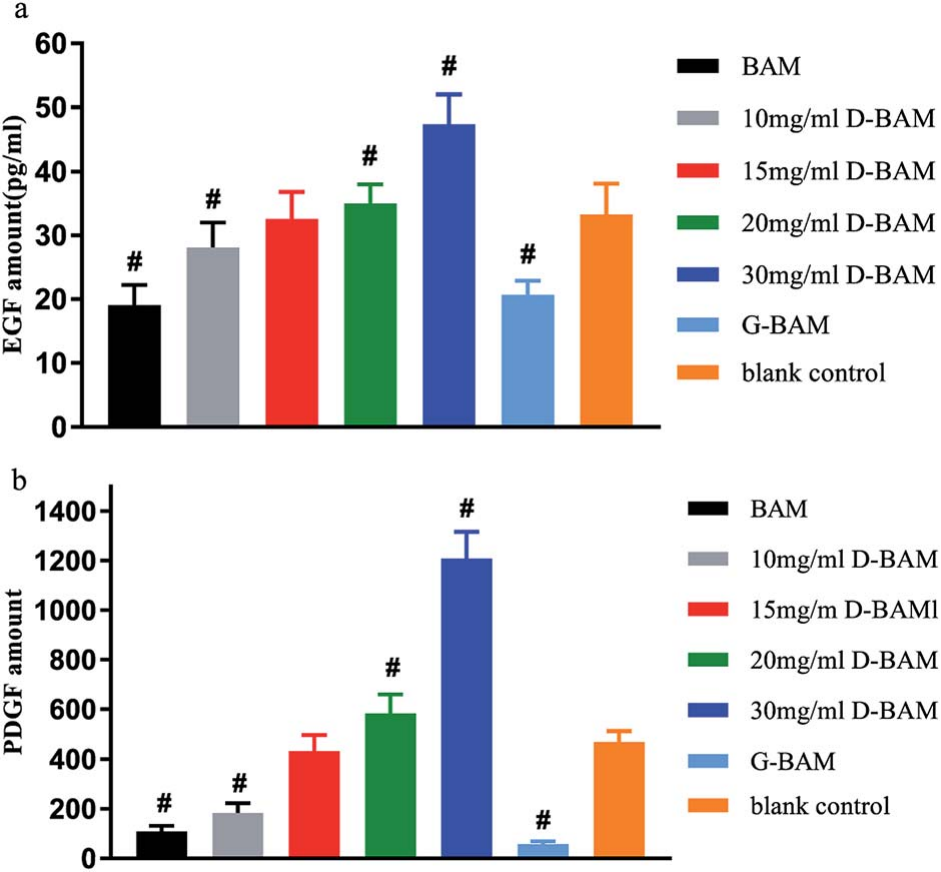
The effect of BAM, GA-fixed and various concentration DCMC-fixed BAM tissues on (a) EGF and (b) PDGF secretion from BTECs. *n* = 3, means ± SD, # means *p* < 0.05 compared with blank.

The secretion of EGF and PDGF protein from BTECs in each group was measured by ELISA assay and presented in Fig. 11. As shown in Fig. 12, the amount of EGF and PDGF was least in the GA-fixed group, while the amount of EGF and PDGF when co-cultured with the 30 mg ml^−1^ DCMC-fixed group was obviously higher, compared to the other counterparts. This result demonstrated that 30 mg ml^−1^ D-BAM tissues could significantly enhance the secretion of EGF and PDGF protein from attached BTECs. This result occurred because of the following aspects: first, due to very low cytotoxicity, and the special biological functions possessed by DCMC at concentration of 30 mg ml^−1^, 30 mg ml^−1^ D-BAM tissues could obviously promote BTECs proliferation and growth; thus, there were more BTECs on BAM tissues that could secrete EGF and PDGF protein. Second, we inferred that EGF and PDGF secreted into medium might have synergistic effects. EGF might increase the secretion of PDGF from BTECs through paracrine action, and conversely, the secretion of PDGF in BTECs might also promote EGF secretion. These findings suggested that BAM tissues fixed by 30 mg ml^−1^ DCMC had a potential ability to promote re-epithelialization, which was very important for the substitute for the bladder.

## 3. Materials and methods

### 3.1 Preparation of DCMC

10.0 g carboxymethyl cellulose sodium was dissolved in 200 ml deionized water and stirred for hours to get a clear solution. Then 100 ml sodium periodate solution was added under continuously magnetic mechanical stirring at room temperature in darkness for hours. Excess ethylene glycol was used to decompose the remaining periodate. The DCMC was obtained after filtration, purification and lyophilization. DCMC with different degrees of oxidation was generated by using different concentrations of sodium periodate solution (the mass ratio of NaIO_4_/CMC is 1 : 1, 1.1 : 1, 1.3 : 1) and different reaction time (2 h, 4 h and 7 h). The value of oxidation degree (OD) was evaluated as following formula according to hydroxylamine hydrochloride/sodium hydroxide method:
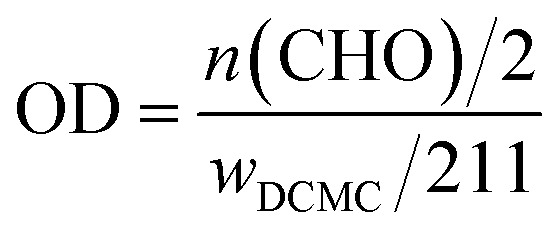
where 211 is approximately the molecular weight of repeating unit in DCMC. Experiments were performed in triplicate.

### 3.2 FITR spectroscopy

FTIR spectra were recorded to characterize the chemical structure of native cellulose and DCMC. 2 mg of dry sample and 20 mg potassium bromide (KBr) were pressed into a disc. The FTIR spectroscopy was performed by a Nicolet 560 under the resolution of 4 cm^−1^ in the wave number ranging from 400 cm^−1^ to 4000 cm^−1^.

### 3.3 DCMC cytotoxicity test

DCMC with different OD generated by using different concentrations of sodium periodate solution and different reaction time were dissolved in DMEM medium (supplemented with 10% fetal bovine serum, 10 mM HEPES, 100 units per ml penicillin, and 100 μg ml^−1^ streptomycin) to make a concentration of 400 μg ml^−1^ DCMC sample solution. P3 generation L929 fibroblasts were seeded in 96 well cell culture plates at a cell density of 5 × 10^4^ per ml, and 100 μl of cell suspension was added to each well. 100 μl of 400 μg ml^−1^ DCMC sample solution was also added to each well in the treatment group, and 100 μl of DMEM medium was added to each well in the control group. The cell culture was maintained at 37 °C with 5% CO_2_. MTT assay was carried out at 1 d, 3 d, and 5 d. The cytotoxicity was expressed as the percentage reduction of cell viability in terms of relative growth ratio (RGR) and calculated with the following formula: RGR = experimental group absorbance value/control group absorbance value × 100%.

### 3.4 Preparation of BAM

The fresh urinary bladder of pig was procured from the local abattoir and immediately preserved in cold physiological saline. The tissues were rinsed with normal saline to remove the adhered blood. The maximum time period between the retrieval and the initiation of protocols was less than 6 h. Then the porcine bladder tissues were pretreatmented by freeze-drying at −42.9 °C for 5 h. After that, the lyophilized bladder tissues were placed in a PBS buffer containing 0.4% w/v ethylene diamine tetra-acetic acid (EDTA) and 0.25% trypsin for 24 h at 37 °C with constant stirring, followed by 1% solution of octylphenoxypolyethoxyethanol (Triton X-100) in PBS buffer for another 24 h at room temperature with constant stirring. Finally, the sample-tissues were digested in 0.02 mg ml^−1^ RNaseA enzyme solution and a 0.2 mg ml^−1^ DNaseI enzyme solution at room temperature overnight to completely remove residual cellular components.

### 3.5 The detection of residual DNA, antigen and growth factors of BAM

The test-samples were pre-frozen in a −80 °C refrigerator for 24 h, and then lyophilized with a vacuum freeze dryer. Adequate lyophilized-samples were weighed and placed in a sterile 1.5 ml microcentrifuge tube. The DNA was then isolated from the samples using the DNeasy™ (Qiagen, Valencia, CA) kit. The total DNA content was quantified by photometric measurement of the optical density at 280 nm. Fresh bladder tissues and BAM samples were examined immunohistochemically by α-SMA staining and α-Gal staining for immunogenicity of tissues.

The content of glycosaminoglycans (GAGs), human keratinocyte growth factor (KGF) and transforming growth factor beta (TGF-β) proteins in the sample-tissues were detected by ELISA following the kit's experimental procedure.

### 3.6 Crosslinking and characterization of D-BAM

#### 3.6.1 Cross-linking of BAM by DCMC

The decellularized BAM samples were washed with PBS solution and cut into the desired size and shape. And then BAM samples were crosslinked by soaking in DCMC/PBS solution (pH 7.4) with vary concentrations of DCMC (10, 15, 20 and 30 mg ml^−1^) at 37 °C for 3 days with constant stirring. Meanwhile, the BAM samples fixed by 0.625% glutaraldehyde (GA) solution were served as control. To reduce reverse reaction, samples were reimmersed in 1.0 wt% NaBH_4_/ethanol solution at 4 °C for 3 h with continuous agitating, and then washed with saline for several times to remove residual inorganic salt and followed by crosslinking characteristics tests.

#### 3.6.2 Characterization of D-BAM

##### 3.6.2.1 Fixation index determination

After being fixed for predetermined time, all BAM samples were lyophilized to a constant weight. Then BAM samples were placed in NHN solution and heated to boil for 20 min. Optical absorbance of the solution was measured by a 722S visible spectrophotometer at 570 nm. Glycine at various known concentrations was used as standard and FI was calculated as following formula:
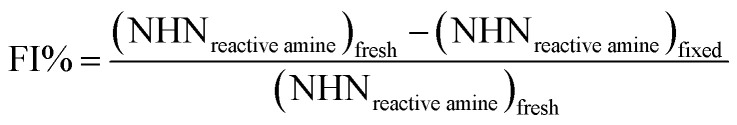


##### 3.6.2.2 Biomechanical test

The mechanical properties of BAM samples, GA-fixed and DCMC-fixed BAM samples (*n* = 5) was examined to determine improvement of their biomechanical property after fixation. BAM samples were cut into a rectangular strip with a length of 40 mm and a width of 5 mm. The accurate width and thickness of each sample was measured using a micrometer. The biomechanical test of each sample was operated on Instron material testing machine (Instron Co., USA) at extension rate of 5 mm min^−1^. After test, the ultimate tensile strain and the ultimate tensile stress were recorded before failure. The ultimate elastic modulus was determined from the stress–strain curves. During testing, the samples were kept in air.

##### 3.6.2.3 Morphologic observation

The BAM samples, G-BAM and D-BAM samples were observed using light microscopy. The samples were fixed in 4% formaldehyde for 72 h, followed by hematoxylin & eosin (H&E) staining for observing the total framework and structure integrity of the samples in the ultrastructure. Meanwhile, the samples were also tested histologically by Masson staining for collagen fibers.

##### 3.6.2.4 The effect of DCMC on the antigen and growth factors of BAM

The content of glycosaminoglycans (GAGs), human keratinocyte growth factor (KGF) and transforming growth factor beta (TGF-β) proteins in the D-BAM were examined by ELISA. α-SMA staining and α-Gal staining were also carried out for determining immunogenicity of D-BAM.

##### 3.6.2.5 *In vitro* enzymatic degradation

To evaluate the capacity of enzymatic hydrolytic resistance of D-BAM, *in vitro* enzymatic degradation was carried out according to a previously reported method. Since collagen fibers are the most important component of the framework structure of animal derived tissues. Therefore, collagenase type I with an activity of 125 U mg^−1^ solid was used for digestion of samples. After being lyophilized and weighted, samples were placed in 250 U ml^−1^ collagenase/PBS solution at 37 °C for different periods (0.5 h, 1 h, 3 h, 6 h, 12 h, 24 h) under c continuous shaking. The enzymatic hydrolysis was stopped by adding 50 μl 10 mM EDTA solution. The dry weight of lyophilized residuals was remeasured. The weight of the sample before degradation was recorded as *W*_0_, and the weight after degradation was recorded as *W*_t_. The weight loss percentage (*W*%) was calculated as following formula: Δ*W*% = (*W*_0_ − *W*_t_)/*W*_0_ × 100%.

##### 3.6.2.6 Anti-calcification test

To evaluate the *in vitro* anti-calcification properties of D-BAM, a simulated body fluid (SBF) assay was used. All the samples were immersed in SBF solution (10 ml per piece) at 37 °C for 7 days under continuous shaking. Subsequently, all samples were removed and washed several times with D-Hanks solution for 1 h to eliminate soluble calcium adsorbed in tissues, and then they were naturally air-dried for EDS analysis.

### 3.7 Cytocompatibility of D-BAM

The effects of decellularized and crosslinked BAM on cell adhesion and proliferation were evaluated using bladder transitional epithelial cells (BTECs). BTECs were purchased from the Cryopreservation Cell bank of West China Hospital. Cell proliferation on scaffolds was measured with MTT test and the distribution and morphology of BTECs grown on the surface were examined by using the SEM. BTECs were cultured in DMEM supplemented with 10% fetal bovine serum, 100 U mL^−1^ penicillin and 100 U mL^−1^ streptomycin within a humidified atmosphere (37 °C, 5% CO_2_ in 95% air). The culture mediums were replaced every other day. Cells from passage 3 were employed for further experiment. For cell proliferation test, after sterilized samples were put into a 24-well plate, 200 μl medium at a density of 1 × 10^5^ cells per ml and 300 μl DMEM medium were used to seed the samples kept in 24 well plates, followed by culturing at 37 °C for different periods (2, 4 and 6 days). At selected time points, the BTECs proliferation was evaluated by MTT test according to the manufacturer's instruction. After BTECs were seeded on sterilized samples and continuously incubated for 4 days, their attachment morphology was observed using scanning electron microscopy (SEM).

### 3.8 Epidermal growth factor (EGF) and platelet derived growth factor (PDGF) protein secretion

The EGF and PDGF protein levels in the culture-medium were evaluated through ELISA. Various samples with attached cells were prepared as detailed above. After the samples were co-cultured with BTECs for 7 days, their supernatant liquid was collected and centrifuged at 14 000 rpm for 5 min for ELISA assay. A standard curve was plotted according to the measured value of a series concentration of EGF & PDGF standard solution. Then, the ELISA assay was performed according to the manufacturer's instructions (R&D Corp.) to determine accurate concentrations of EGF and PDGF, and their values were expressed as pg ml^−1^.

## 4. Conclusion

In summary, the results in our study implied that DCMC could be used as effective crosslinking reagent for BAM tissue fixation and the D-BAM tissues were suitable to used as a substitute for the bladder. For the preparation of DCMC, the results showed that when DCMC was prepared using a specific concentration of sodium periodate solution (the mass ratio of NaIO_4_/CMC is 1 : 1) and a specific reaction time (4 hours), its cytotoxicity was the smallest and its fixation effect was better. Compared with the other experimental groups, we find that BAM tissues fixed with 30 mg ml^−1^ DCMC exhibited best crosslinking characteristics. DCMC fixation significantly improved biomechanical strength of BAM tissues and its resistance to enzymatic degradation. Meanwhile, the original ultrastructure of the BAM tissues was well preserved after DCMC fixation. The DCMC-fixation could also reduce the expression of α-Gal in BAM tissues and preserve the useful growth factor such as GAGs, KGF and TGF-β in bladder tissues. Moreover, the D-BAM tissues (especially fixed by 30 mg ml^−1^ DCMC) were characterized to be low cytotoxicity and low antigenicity. It could also stimulate the secretion of PDGF and EGF from seeded BTECs, which is a critical feature for further re-epithelialization. What is more, DCMC fixation (especially 30 mg ml^−1^ DCMC-fixation) significantly inhibited mineral deposits forming within BAM tissues than GA fixed control group, which indicated the potential good anti-calcification ability.

## Conflicts of interest

There are no conflicts to declare.

## Supplementary Material

RA-009-C9RA07965C-s001
