# Supplementary material for: Development and characterization of bladder acellular matrix cross-linked by dialdehyde carboxymethyl cellulose for bladder tissue engineering
Source: RSC Adv. 2019 Dec 18;9(72):42000–9. doi: 10.1039/c9ra07965c (PMC9076556; doi:10.1039/c9ra07965c)
Supplement: RA-009-C9RA07965C-s001 [file RA-009-C9RA07965C-s001.pdf]

Electronic Supplementary Material (ESI) for RSC Advances.
This journal is © The Royal Society of Chemistry 2019

Supplementary material

Supplementary Results

Molecular weight and distribution of DCMC

Periodate can oxidize 1,2-dihydroxy of CMC to aldehyde groups under acidic conditions, which is highly specific and has no obvious adverse reactions. However, there is a degradation in the process of preparing the aldehyde derivatives of CMC by periodic acid oxidation, resulting in decrease of crystallinity. Therefore, it is necessary to discuss the molecular weight of the DCMC crosslinker. With the increasing of concentration of sodium periodates used in preparation of DCMC and prolonging of reaction time for preparation of DCMC, the polysaccharide chain was broken, and the Mn and Mw of DCMC presented a downward trend. As we know, the shorter the reaction time for preparation of DCMC leading to incomplete reaction was, the bigger the molecular weight of DCMC was. On the contrary, the longer the reaction time or the higher the concentration of sodium periodates used in preparation of DCMC was, the smaller the molecular weight of DCMC was. DCMC with high molecular weight has low cytotoxicity and low crosslinking property, while DCMC with small molecular weight has high cytotoxicity and high crosslinking property. So the key to preparing DCMC is to select appropriate reaction time and amount of sodium periodate. (Table 1.)

Table.1 Molecular weight and distribution of DCMC

$NaIO_4$/CMC	Reaction time	Mn	Mw	Mw / Mn
1:1	2h	124,750	283,679	2.274
	4h	80,616	184,039	2.283
	7h	38,551	94,964	2.463
1.1:1	2h	70,999	248,259	3.497
	4h	33,146	185,882	5.608
	7h	20,324	68,291	3.360
1.3:1	2h	66,157	145,340	2.197
	4h	45,999	139,920	3.042
	7h	11,269	17,671	1.568
